# Ethnic Specificity of Species and Strain Composition of *Lactobacillus* Populations From Mother–Infant Pairs, Uncovered by Multilocus Sequence Typing

**DOI:** 10.3389/fmicb.2022.814284

**Published:** 2022-03-04

**Authors:** Lixia Yuan, Xueling Zhang, Baolong Luo, Xu Li, Fengwei Tian, Wenli Yan, Yongqing Ni

**Affiliations:** ^1^School of Food Science and Technology, Shihezi University, Shihezi, China; ^2^School of Food Science and Technology, Jiangnan University, Wuxi, China

**Keywords:** *Lacticaseibacillus paracasei*, vertical transmission, multilocus sequence typing, ethnic specificity, species composition

## Abstract

The maternal gut is thought to be the principal source of potential probiotic bacteria in the infant gut during the lactation stage. It is not clear whether facultative symbiont lactobacilli strictly follow vertical transmission from mother to infant and display the ethnic specificity in terms of species and strain composition in mother–infant cohorts. In the present study, a total of 16 former *Lactobacillus* species (365 strains) and 11 species (280 strains) were retrieved from 31 healthy mother–infant pairs of two ethnic groups, which have never intermarried, respectively. The result showed that the composition and number of *Lactobacillus* species between the two ethnic groups varied. Among 106 *Lacticaseibacillus paracasei* strains isolated, 64 representative strains were classified into 27 sequence types (ST) by means of multilocus sequence typing (MLST), of which 20 STs derived from 33 Uighur strains and 7 STs from 31 Li strains, and no homologous recombination event of genes was detected between strains of different ethnic groups. A go-EBURST analysis revealed that except for a few mother–infant pairs in which more than one STs were detected, *L. paracasei* isolates from the same mother–infant pair were found to be monophyletic in most cases, confirming vertical transfer of *Lactobacillus* at the strain level. More notably, *L. paracasei* isolates from the same ethnic group were more likely than strains from another to be incorporated into a specific phylogenetic clade or clonal complex (CC) with similar metabolic profile of glycan, supporting the hypothesis of ethnic specificity to a large degree. Our study provides evidence for the development of personalized probiotic tailored to very homogenous localized populations from the perspective of maternal and child health.

## Introduction

The human gut represents a complex ecosystem that is colonized by trillions of microorganisms which influence the physiology, immune function, and health status of the host (Martin et al., 2016). Accumulating evidence highlights that the maternal gut serves as the most relevant source of bacteria that are detectable in breast milk, and the breast milk acts as an intermediary for the transfer of potential probiotics from mother to infant, especially the members of the former genera *Lactobacillus* and *Bifidobacterium* (McGuire and McGuire, 2017; Sindi et al., 2021). Indeed, multiple recent studies have showed the sharing of specific strains of *Bifidobacterium* species between microbiomes in the mother–breast milk–infant triad, strongly indicating vertical transmission from mother to child.

Like *Bifidobacterium*, *Lactobacillus* was also recognized as one of the key contributors to beneficial effects for maternal–infant health by providing an inoculum of *Lactobacillus* to the infant gut. For example, some members of the former genus *Lactobacillus* play an important role in regulating human metabolism and immune system (Abbasi et al., 2021). Additionally, *Lactobacillus* are considered as the key elements of the defensive system in the infant’s intestines (Solís et al., 2010), which inhibits a wide spectrum of infant pathogenic bacteria by competitive exclusion or production of antibacterial compounds (Olivares et al., 2006). For example, *Lactobacillus* GG and lactoferrin could enhance the antibacterial defense of the neonatal intestinal epithelium (Sherman et al., 2004).

Based on traditional culturing, the main taxa phylogenetically affiliated with the former genus *Lactobacillus* reported from adult gut (maternal cohort) were so far *Lactobacillus gasseri*, *Lacticaseibacillus rhamnosus*, *Lactobacillus jensenii*, *Lactobacillus iners*, *Lacticaseibacillus casei*, *Lacticaseibacillus paracasei*, *Ligilactobacillus salivarius*, *Limosilactobacillus fermentum*, *Lactobacillus crispatus*, and *Lactiplantibacillus plantarum* (Walter, 2008; Murphy et al., 2017; Ding et al., 2019; Juliana et al., 2021). Also, the infant-associated *Lactobacillus* species were *L. fermentum*, *L. rhamnosus*, *Lactobacillus brevis*, *Lactobacillus reuteri*, and *L. plantarum* (Moeller et al., 2016; Zhang et al., 2020). According to published literature, there are more *Lactobacillus* species reported in human gut than *Bifidobacterium* spp. Although *Lactobacillus* species were scarcely reported in human skin specimens using either classical plating techniques and/or DNA sequencing-based approaches, human-associated *Lactobacillus* species exist not only in the human breast milk and gut tract but also in the oral cavity and vagina of healthy mothers (Martín et al., 2007; Nadkarni et al., 2020; Wang et al., 2021). Given lifestyle transitions of some *Lactobacillus* species from free living to host adapted, the migration of some exogenously free-living *Lactobacillus* species to the infant’s gut cannot be ruled out. Therefore, it is necessary to pay special attention to whether human gut-residential *Lactobacillus* strictly follow vertical transmission from mother to child and/or play a role in seeding in the infant gut during lactation.

Humans have co-evolved with their gut microbiota for millions of years, leading to the development of symbiosis between humans and some intestinal microbes, such as *Bifidobacterium* spp. (Essock-Burns et al., 2021). Meanwhile, due to sub-population migration and isolation by distance (Spurgin et al., 2014), human evolution has been accompanied by periodic fundamental changes in lifestyle reflected in their dietary habits (He et al., 2018). Clearly, the human–microbe symbiosis is based on the reciprocity of nutrient metabolism of carbohydrate in the daily diet (Colman et al., 2012; Rook et al., 2017; Brinker et al., 2019). Human and model animal studies have shown the role of host genetics in shaping both the overall microbiome composition and the individual bacterial taxa (Cahana and Iraqi, 2020). Based on the above two points, human diet and genetics are likely to exert host selectivity on the gut microbiome composition from the community to species level and even strain level. Particularly, in contrast to *Bifidobacterium* spp., such as *Bifidobacterium bifidum*, *Bifidobacterium breve*, and *Bifidobacterium longum* ssp. *infantis*, most *Lactobacillus* species in the human intestinal tract are not obligative endosymbionts but facultative symbionts (Martino et al., 2018). Host adaptation of *Lactobacillus* species is associated with genomic events that are characteristic of the evolution of a symbiotic lifestyle (Frese et al., 2011; Papizadeh et al., 2017). So different strains of the same *Lactobacillus* species showed obvious differences in glycan metabolism and different host adaptation.

More recently, the *gro*EL gene proved to be a more effective target for the identification of *Lactobacillus* species through amplicon sequencing than 16S rRNA gene (Zuñiga et al., 2017; Yang et al., 2019). In the present study, we isolated and distinguished *Lactobacillus* strains from 31 healthy mother–infant pairs of two ethnic groups, including Xinjiang Uighur and Hainan Li of China, the habitats of which are separated by more than 4,000 km, with geographical and climatic environments being greatly different. For thousands of years, the two ethnic groups rarely intermarry, and their dietary customs and foodstuffs vary greatly. The purpose of our study was to illuminate weather lactobacilli strictly follow vertical transmission from mother to infant. To achieve this goal, we determined the concordance of the multilocus sequence types (ST) of strain collective of the dominant *L. paracasei* (former *Lactobacillus paracasei*, abbreviated as *L. paracasei*) shared by mother–infant pairs of the same cohorts. Also, another objective was to provide evidence for the development of personalized probiotic tailored to a very homogenous sub-population from the perspective of maternal and child health, by seeking ethnic specificity in the composition of *Lactobacillus* species and strain in different mother–infant cohorts.

## Materials and Methods

### Subjects and Sample Collection

We recruited 31 mothers and their infants (6 months old) from October 10 to December 26, 2019, at Hetian, Xinjiang, and Changjiang, Hainan, China. Mother–infant pairs were recruited with the following inclusion criteria: (1) volunteers had no recent history of gastrointestinal complaints; (2) not taking antibiotics or drugs that affect gut microbes for at least 6 months before or after delivery; and (3) not taking probiotic products. Our study was conducted according to the guidelines of the Declaration of Helsinki. In addition, all procedures involving human subjects were then adopted by the Ethics Committee of the First Affiliated Hospital, Shihezi University School of Medicine (2017-117-01).

After cleaning the nipple and areola with a cotton swab in sterile water and discarding the first few drops, breast milk samples (3–5 ml) were collected into sterile collection tubes by manual expression using sterile gloves. Fresh fecal samples of mother and infant approximately 10–15 g were collected from the feces of the participants and stored in sterile collection tubes. After collection, the sampling tube was labeled. All collected bacterial samples were temporarily stored in the vehicle refrigerator at −20°C and would be transported back to the laboratory for bacterial separation within a week.

### Isolation of *Lactobacillus* on Selective Medium

Subsamples of breast milk (1–2 ml) and fecal sample (5–10 g) were immediately selected for the separation of *Lactobacillus*. The breast milk sample was diluted to 10^–3^ with sterile saline; the fecal samples were defrosted at room temperature, homogenized by kneading, and 10-fold diluted down to 10^–4^ with sterile saline.

The diluent of 200 μl feces samples was coated on the modified Man–Rogosa–Sharpe (MRS) agar medium (add 20 mg vancomycin hydrochloride per liter) and LAMVAB selective agar medium (Hartemink et al., 1997) of *Lactobacilli* then anaerobically cultured at 37°C for 48 h (three replicates for each sample), and the morphology of the bacteria was examined under a microscope. Fifteen to 20 single colonies with different colony morphology were selected from each medium plate, and 30–60 colonies were obtained from each sample. After three culture passages, the positive Gram staining and negative catalase were initially identified as suspected *Lactobacillus* (Klayraung and Okonogi, 2009; Jomehzadeh et al., 2020), and the pure culture was stored in 25% glycerol at −80°C.

### Genomic DNA Extraction and DNA Fingerprinting

Genomic DNA was extracted from each of the fresh colonies as introduced by Duckchul Park, with slight modification (Park, 2007). Next, unidentified Gram-positive rod-shaped *Lactobacillus* isolates from all samples were initially screened and grouped using repetitive PCR (rep-PCR) fingerprints for cost-effective speciation and typing. Then, rep-PCR reaction was performed using the BoxAIR (5′-CTACGGCAAGGCGACGCTGACG-3′) primer and (GTG)_5_ (GTGGTGGTGGTGGTG) primer. Each 25 μl PCR sample contained 12.5 μl Premix Taq, 1 μl primer (20 μM) and 8.5 μl ddH_2_O. Amplification for BoxAIR utilized an initial denaturation step of 94°C for 30 s followed by 35 cycles of 94°C for 30 s, annealing at 52°C for 30 s, extension at 72°C for 4 min, and a final step of 65°C for 10 min. The annealing temperature of (GTG)_5_ was 51°C.

Amplified products were separated by horizontal electrophoresis on a 1.8% (w/v) agarose gel in 0.5 × Tris-acetate-EDTA (TAE) buffer at 100v for 1.5 h. The results were observed and photographed in an ultraviolet gel imager (QUANTUM ST5 Vilber Lourmat, France). The comparison of rep-PCR banding patterns was performed using the computer software package GelCompar II v6.0 (Applied Math, Sint-Martens-Latem, Belgium). The Pearson similarity coefficient was calculated, and the unweighted pair group method with arithmetic mean (UPGMA) was used for grouping. Finally, at least one representative strain was selected from each group for sequencing analysis.

### Identification of the Bacterial Isolates

The isolates were identified at the species level PCR sequencing of the *groEL* gene by using the primers Lac-*groEL*-F (5′-TCCGATTACGAYCGYGAGAAGCT-3′) and Lac-*groEL*-R(5′-CSGCYTCGGTSGTCAGGAACAG-3′) (Hu et al., 2017). The obtained sequences were submitted to GenBank database, and BLAST^1^ was used for sequence homology analysis to compare the similarity between test strains and corresponding sequences of known *Lactobacillus* strains. A phylogenetic tree was constructed using the p-distances and Kimura-2 parameter distances of MEGA 6.0 software and subjected to 1,000 bootstrap tests (Saitou and Nei, 1987; Tamura et al., 2013).

### DNA Fingerprinting of *Lacticaseibacillus paracasei*

A total of 106 DNA of *L. paracasei* strains were used for BoxAIR and (GTG)_5_ rep-PCR assay (data not provided). The amplification conditions were the same as those in fingerprint typing mentioned above. Through cluster analysis, samples with different fingerprint band and from different mother–infant pairs were screened for follow-up experiments, and a total of 64 representative strains based on BoxAIR and (GTG)_5_ primers were obtained (Supplementary Figure 1).

### Housekeeping Gene Primer Selection

Internal amplified fragment from seven housekeeping genes (*recA*, *rlpB*, *fusA*, *lepA*, *ileS*, *recG*, *pyrG*) were amplified using primers listed in Table 1. The primers used in this experiment were by Biotechnology GENEWIZ (JiangSu). PCR amplifications were referenced with the methods described by Diancourt et al. (2007). PCR amplification conditions included pre-denaturation at 94°C for 5 min; 30 cycles of 94°C for 30 s, annealing at 50–55°C (optimal annealing temperatures for each locus are listed in Table 1) for 30 s, extension at 72°C for 30 s, and finally at 72°C for 5 min.

**TABLE 1:** Housekeeping genes and primer information for MLST of *L. paracasei.*

Gene	Enzyme function	Primer	Sequence (5′–3′)	Size (bp) of analyzed fragment	Annealing temperature (°C)
*pyrG*	CTP synthase	*pyrG*-F	5′-GGGGTCGTATCGTCATTGGGTAAAGG-3′	345	55
		*pyrG*-R	5′-GGAATGGCAATGATTCGTATCGCCAA-3′		
*fusA*	Protein elongation factor EF-2	*fusA***-**F	5′-CCGTAATATCGGGATCATGGCTCACATCGA-3′	663	55
		*fusA*-R	5′-CAACAACATCTGAACACCCTTGTT-3′		
*iIeS*	Isoleucyl-tRNA synthase	*ileS*-F	5′-TCCTGGTTGGGATACTCACGG-3′	360	55
		*ileS*-R	5′-AGGAACCGGAATCGAACCACACATC-3′		
*IepA*	GTP-binding protein LepA	*lepA*-F	5′-CATCGCCCACATTGATCACGGGAA-3′	549	55
		*lepA*-R	5′-CATATGCAGCAAGCCTAAGAACCC-3′		
*recG*	ATP-dependent DNA helicase	*recG*-F	5′-AGGCGATGTTGGGAGCGGTAAAAC-3′	342	51
		*recG*-R	5′-GTGTTCGGGGAATAGGCGTCGC-3′		
*recA*	Recombinase A	*recA*-F	5′-CCGGAAAGTTCCGGCAAAACAAC-3′	315	50
		*recA*-R	5′-CGCGACCACCTGGTGTCGTTT-3′		
*rlpB*	50S ribosomal protein L2	*rlpB*-F	5′-CAACAGTTAAAGCAATCGAATACGATCC-3′	366	55
		*rlpB*-R	5′-CACCACCACCATGCGGGTGATC-3′		

### Experiment on Polysaccharide Utilization

*Lactobacillus* strains were cultured anaerobically at 37°C for 48 h on MRS agar medium. Strains were passaged to MRS liquid media; after 48 h, cultures were inoculated in liquid MRS medium with 2% inoculation capacity and anaerobically cultured at 37°C with different carbohydrate-supplemented media. The strains were also grown on MRS liquid media without carbon source as blank control, and glucose as carbon source was used as positive control. After 48 h, 100 ml of each culture was transferred to a flat-bottom 96-well plate, and OD600 nm was measured with Envision plate reader. The final OD600 nm value for each oligosaccharide condition was calculated by subtracting the mean blank OD600 nm from the OD600 nm for each strain grown in the corresponding oligosaccharide. Each experiment was repeated three times.

### Data Analysis

The resulting gel images were analyzed with the software package GelCompar II v6.0. The utilization of polysaccharides in *L*. *paracasei* was analyzed by R (version 4.0.4). Sequence data of multilocus sequence typing (MLST) were edited using Chromas 2.4.1 software and then aligned in MEGA6.0 software using CLUSTAL W. Analyses of allele sequences and MLST were performed using BioNumerics software V8.0 (Applied-Maths, Sint Maartens-Latem, Belgium) by the UPGMA and categorical coefficient of similarity. Minimum-spanning tree analysis was constructed with Prims’s algorithm embedded in the BioNumerics software according to isolate source and ethnic group. The number of alleles, G + C content, dN/dS ratio, and Tajima’s *D*-value were calculated with the software DnaSp v 5.1. Split-treev4.0 software was used to evaluate the impact of recombination events on phylogeny, and split decomposition analysis was constructed. A *phi*-test attached to this software was used to detect whether there was significant recombination in alleles. Nucleotide sequences of seven loci were deposited in the GenBank database under accession numbers OM264880 to OM264907.

## Results

### Study Population

In this study, we enrolled 31 mother–infant pairs (Uighur *n* = 17, Li *n* = 14). At the initial study visit, participants were interviewed about some basic clinical data, including the height, weight, and daily diet of the mother and the birth date, weight, sex, and feeding characteristics of the infant. The clinical characteristics and demographic data of the mothers and infants are reported in Supplementary Table 1.

### The Composition of *Lactobacillus* Analysis Based on Culture Method

In order to retrieve the species diversity of *Lactobacillus* as much as possible, two selective media were used for *Lactobacillus* isolation. For breast milk samples, there were low bacterial counts on two selective media (0–3.25 log CFU/ml). Conversely, almost 80% of fecal samples readily yielded colonies, ranging from 3.63 to 6.98 log CFU/ml on modified MRS agar and 2.98–6.02 log CFU/ml on LAMVAB selective medium. According to morphological, biochemical, and physiological testing, a total of 966 isolates (523 isolates from Uighur and 443 isolates from Li) were considered putatively to be lactobacilli. Strains with identical rep-PCR DNA-banding patterns were considered to belong to the same species. The two to three representative isolates of each rep-PCR group were selected for *groEL* gene sequencing. The *groEL* gene sequences were compared to the entries in GenBank using Blastn (see footnote 1), and those displaying identity > 96% with known species were identified as corresponding species. Characterization by fingerprinting allowed 365 representative *Lactobacillus* isolates of 32 rep-PCR groups from Uighur to be assigned to 16 species, and 280 representative *Lactobacillus* isolates of 23 rep-PCR groups from Li to 11 species.

**FIGURE 1:**
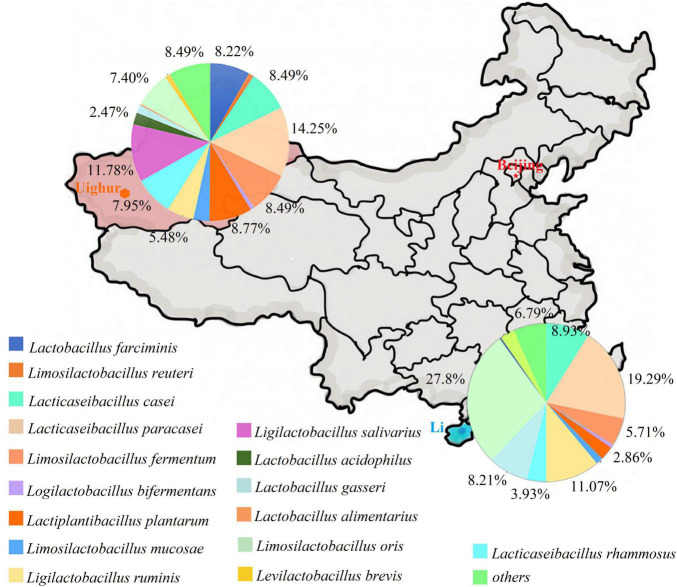
The composition of *Lactobacillus* from mother and infant between Uighur and Li based on culture method. *Lactobacillus* were identified by *groEL* gene sequencing.

By referring to an updated taxonomic revision for the former genus *Lactobacillus* (Zheng et al., 2020), the composition of culturable *Lactobacillus* species in two ethnic groups was listed as shown in Figure 1. The species compositions and proportion of *Lactobacillus* varied depending on the ethnic groups. In Uighur samples, a relatively higher species diversity was observed, with 16 *Lactobacillus* species dominated by *L. paracasei* (14.2%), *L. salivarius* (11.8%), *L. plantarum* (8.8%), *L. casei* (8.5%), *L. fermentum* (8.5%), and *Lactobacillus farciminis* (8.2%). By contrast, 11 *Lactobacillus* species were identified in the Li samples, among which *Limosilactobacillus oris* (27.9%), *L. paracasei* (19.3%), *Ligilactobacillus ruminis* (11.1%), *L. casei* (8.9%), *L. gasseri* (8.2%), and *L. fermentum* (5.7%) were the main species. Of 16 species identified, *L. casei*, *L. paracasei*, *L. fermentum*, *Loigolactobacillus bifermentans*, *L. rhamnosus*, *L. gasseri*, and *L. oris* were the bacterial species shared by the Uighur and Li ethnic groups. We found that some species were commonly present in the three ecosystems (maternal feces, breast milk, and infant feces). Considering the species that appeared in the three ecosystems simultaneously, vertical transmission between mother and infant was likely. The occurrence of *Lactobacillus* in each of the mother–infant pairs are listed in Table 2. The species that Uighur mothers and infants shared in the three ecological niches were *L. casei* (1 pair), *L. paracasei* (1 pair), and *L. salivarius* (2 pairs), while the Li only had *Limosilactobacillus mucosae* (1 pair). *L. paracasei* (2 pairs), *L. salivarius* (1 pair), and *L. plantarum* (1 pair) appeared in Uighur breast milk and infant feces, while only *L. mucosae* (1 pair) appeared in Li. Again, *L. casei* (3 pairs), *L. paracasei* (16 pairs), *L. salivarius* (3 pairs), *L. gasseri* (3 pairs), and *L. oris* (2 pairs) were isolated simultaneously in the maternal and infant feces of the two ethnic groups. Particularly, prevalence of *L. paracasei* was the highest in two ethnic groups. So we selected *L. paracasei* shared by mothers and infants of the two ethnic groups to seek the ethnic specificity of human-associated *Lactobacillus* between different cohorts at the strain level.

**TABLE 2:** The *Lactobacillus* species shared by mother–infant pairs.

Mother–infant pairs	Bacterial species																	
	*Lfar*	*Lreu*	*Lcas*	*Lpca*	*Lfer*	*Lbif*	*Lpla*	*Lmuc*	*Lrum*	*Lrha*	*Lsal*	*Laci*	*Lgas*	*Lali*	*Lori*	*Lbre*	*Lcav*	*Lhar*
T1	* +			# * +	#	#			*		* +				# *	*		
T2	* +		+	+			*				+							
T3	* +										* + #							
T4	#		* +	* +				*	*	+					+			
T5								+	*	*	# * +							
T6	+			* +	+		*	+			+	*	+			#		
T7	* +			* +			*			*				#				
T8		+	* +	# +	#				*						*			
T9		+		* +								*						
T10			# *	* +			+		*		*	+						
T11			*	* +	*	*					*						*	
T12				* +	*		*	*		* +			+		*			
T13			# * +		#		*		*		*	*	* +					
T14								*	*						* +	*		
T15				*	+						*							
T16	+		+	* +						* +					+			
T17	* +		#	# +	#		+ #				# +	#	+		#		*	
N1			+					*					* +					
N2				* +			* +								*			
N3				* +			*	+					*					+
N4								*			* +							
N5				* +	#			# * +		+			* +					+
N6			*	* +		*										+		
N7			*	* +											* +			
N8								*		# *				*			+	
N9			*	* +			*							+				
N10				#	*	*	#											
N11			*	*				*					#		#			
N12				* +	*		+	# +					#	+				
N13			* +	#					+					*	#			
N14				* +	*	+		*				+		+				

Lfar, L. farciminis; Lreu, L. reuteri; Lcas, L. casei; Lpca, L. paracasei; Lfer, L. fermentum; Lbif, Lactobacillus bifermentum, Lpla, L. plantarum; Lmuc, L. mucosae; Lrum, L. ruminis; Lrha, L. rhamnosus; Lsal, L. salivarius; Laci, L. acidophilus; Lgas, L. gasseri; Lali, Lactobacillus alimentarius; Lori, L. oris; Lbre, L. brevis; Lcav, Lactobacillus caviae; Lhar, Lactobacillus harbinensis. #, The bacterial species were only present in the breast milk sample. *, The bacterial species were only present in the maternal feces sample. +, The bacterial species were only present in the infant feces sample, T, represents the strains from Hetian; N, represents the strains from Changjiang.

### Analysis of Multilocus Sequence Typing

Seven conserved gene loci (*recA*, *rlpB*, *fusA*, *lepA*, *ileS*, *recG*, *pyrG*) were amplified by PCR and DNA sequenced. As shown in Table 3, the number of alleles of seven housekeeping genes was 2 (*iIeS* and *pyrG*) and 8 (*rlpB*). The G + C% of the seven housekeeping genes was 46.9–52.8% close to the G + C content of the reference strain *L. paracasei* ATCC334 (46.6%). The average pairwise nucleotide diversity per site among seven genes ranged from 0.00008 to 0.146. The dN/dS ratio of the genes *pyrG*, *recG*, and *recA* was less than 1, suggesting that these housekeeping genes had been subjected to environmental selection pressure and maintained a high degree of conservatism or environmental elimination during evolution. On the contrary, the dN/dS ratios of four other genes were higher than 1, indicating that almost half of genes were selected by external selection pressures which interfered too much for these genes and accelerated their evolution (Zhang et al., 2015). Additionally, the values from Tajima’s *D*-test, which measures deviation from the standard neutral model of evolution, ranged from -2.87887 to -0.79564.

**TABLE 3:** MLST allele sequence information and diversity.

Gene	No. of alleles	G + C%	dS	dN	dN/dS	Tajima’s *D*-value	π	*phi*-test
*pyrG*	2	48.4	0	0.00012	0	–1.43583	0.00017	0
*fusA*	5	52.8	0.02913	0.03301	1.133196	–2.87887	0.02964	1.0
*iIeS*	2	46.9	0.0019	0.0045	2.3684210	–0.79564	0.00343	0
*IepA*	7	46.9	0.08918	0.14189	1.591052	–2.18339	0.146	0.1074
*recG*	4	48.7	0.0515	0.05094	0.989126	–2.78715	0.0564	0
*recA*	3	49.1	0	0.00011	0	–1.07704	0.00008	0
*rlpB*	8	47.5	0.02237	0.03558	1.59052	–2.71703	0.03321	0.4865

*π, nucleotide diversity.*

### Analysis of Sequence Types and Clonal Complexes

**FIGURE 2:**
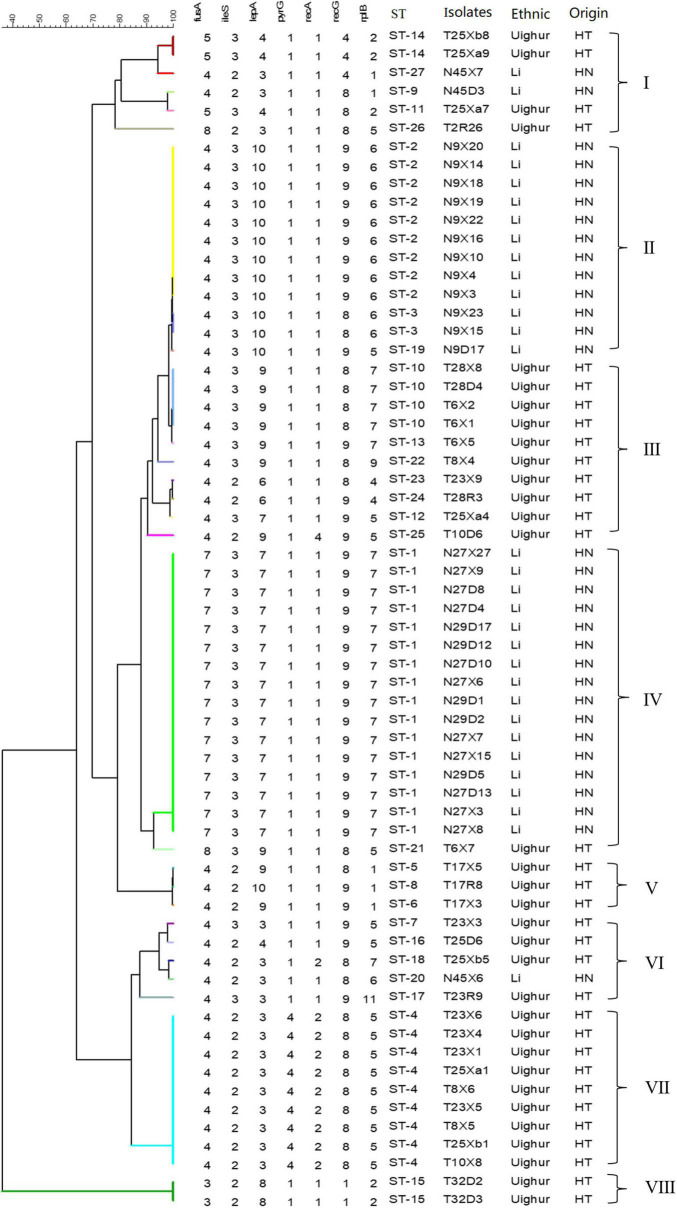
An UPGMA dendrogram of ST of 64 representative *L*. *paracasei* strains. The different colors represent the different STs. Each row shows in turn the number of alleles, ST, and isolated location for each strain.

The sequences obtained for seven housekeeping genes were analyzed by BioNumerics V8.0 software to determine the allele number. The number of alleles in each strain was connected in sequence to form the allelic profiles. The allelic combination of seven gene fragments of 64 representative strains were conducted, and the ST were identified by the uniqueness of allele combination. The characterization of the diversity of seven housekeeping genes was reflected in the UPGMA dendrogram (Figure 2). Except for a few strains, all strains were divided into eight clusters according to ethnic origin. Six (cluster I, cluster III, cluster V, cluster VI, cluster VII, cluster VIII) and two (cluster II, cluster IV) genotypic clusters were found from Uighur and Li *L. paracasei* strains isolated from mother–infant pairs, respectively. It clearly showed from the cluster diagram that the strains from the same ethnic group were mostly clustered together. Overall, 106 *L. paracasei* were divided into 27 STs based on rep-PCR fingerprint typing results (Table 4). Among the 64 representative *L. paracasei* strains, 33 strains isolated from Uighur were classified into 20 STs and 31 representative strains from Li into 7 STs, which indicated that *L. paracasei* had high genetic diversity.

**TABLE 4:** STs and Allelic profiles of 106 *L*. *paracasei*.

Strain	Source	Rep-PCR	ST	Allele number						
		type^a^		*fusA*	*iIeS*	*IepA*	*pyrG*	*recA*	*recG*	*rlpB*
N45 × 7	Uighur	C (2)^b^	27	4	2	3	1	4	4	1
T25Xa9	Li	F	14	5	3	4	1	2	4	2
T25Xb8	Li	F (1)	14	5	3	4	4	2	4	2
T23R9	Li	A (2)	17	4	3	3	1	1	9	11
N45 × 6	Uighur	C (1)	20	4	2	3	1	4	8	6
T23 × 6	Li	D (1)	4	4	2	3	4	2	8	5
T23 × 4	Li	D	4	4	2	3	4	2	8	5
T23 × 1	Li	D	4	4	2	3	4	2	8	5
T25Xa1	Li	E (2)	4	4	2	3	4	2	8	5
T8 × 6	Li	E	4	4	2	3	4	2	8	5
T23 × 5	Li	D	4	4	2	3	4	2	8	5
T8 × 5	Li	E (1)	4	4	2	3	4	2	8	5
T25Xb1	Li	E (1)	4	4	2	3	4	2	8	5
T10 × 8	Li	E	4	4	2	3	4	2	8	5
T25D6	Li	A	16	4	2	4	4	1	9	5
T25Xb5	Li	B	18	4	2	3	4	2	8	7
T23 × 3	Li	F (2)	7	4	3	3	1	1	9	5
T2R26	Li	E	26	8	2	3	4	4	8	5
N45D3	Uighur	C	9	4	2	3	1	1	8	1
T25Xa7	Li	F (2)	11	5	3	4	4	2	8	4
T28R3	Li	C (1)	24	4	2	6	4	4	9	4
T10D6	Li	C (1)	25	4	2	9	4	4	9	5
T23 × 9	Li	A	23	4	2	6	1	4	8	4
T17 × 5	Li	C	5	4	2	9	1	1	9	1
T17R8	Li	B (3)	8	4	2	10	1	1	9	1
T17 × 3	Li	A (2)	6	4	2	9	1	1	9	1
N27 × 27	Uighur	B	1	7	3	7	1	1	9	7
N27 × 9	Uighur	B	1	7	3	7	1	1	9	7
N27D8	Uighur	C (2)	1	7	3	7	1	1	9	7
N27D4	Uighur	F	1	7	3	7	1	1	9	7
N29D17	Uighur	B	1	7	3	7	1	1	9	7
N29D12	Uighur	A (2)	1	7	3	7	1	1	9	7
N27D10	Uighur	B	1	7	3	7	1	1	9	7
N27 × 6	Uighur	B	1	7	3	7	1	1	9	7
N29D1	Uighur	B	1	7	3	7	1	1	9	7
N29D2	Uighur	C	1	7	3	7	1	1	9	7
N27 × 7	Uighur	C (1)	1	7	3	7	1	1	9	7
N27 × 15	Uighur	B	1	7	3	7	1	1	9	7
N29D5	Uighur	B	1	7	3	7	1	1	9	7
N27D13	Uighur	C (1)	1	7	3	7	1	1	9	7
N27 × 3	Uighur	B	1	7	3	7	1	1	9	7
N27 × 8	Uighur	C	1	7	3	7	1	1	9	7
T6 × 7	Li	A	21	8	3	9	1	1	8	5
T28 × 8	Li	A (2)	10	4	3	9	1	1	8	7
T28D4	Li	A	10	4	3	9	1	1	8	7
T6 × 2	Li	B	10	4	3	9	1	1	8	7
T6 × 1	Li	B (3)	10	4	3	9	1	1	8	7
T6 × 5	Li	B	13	4	3	9	1	2	9	7
N9 × 20	Uighur	F	2	4	3	10	1	1	9	6
N9 × 14	Uighur	H	2	4	3	10	1	1	9	6
N9 × 18	Uighur	E	2	4	3	10	1	1	9	6
N9 × 19	Uighur	F	2	4	3	10	1	1	9	6
N9 × 22	Uighur	F (1)	2	4	3	10	1	1	9	6
N9 × 16	Uighur	F	2	4	3	10	1	1	9	6
N9 × 10	Uighur	F	2	4	3	10	1	1	9	6
N9 × 4	Uighur	C	2	4	3	10	1	1	9	6
N9 × 3	Uighur	C	2	4	3	10	1	1	9	6
N9D17	Uighur	G (3)	19	4	3	10	1	1	9	5
T25Xa4	Li	C (1)	12	4	3	7	1	2	9	5
T8 × 4	Li	A	22	4	3	9	1	1	8	9
N9 × 23	Uighur	G (2)	3	4	3	10	1	4	8	6
N9 × 15	Uighur	F	3	4	3	10	1	4	8	6
T32D2	Li	G (2)	15	3	2	8	4	1	1	2
T32D3	Li	G	15	3	2	8	4	1	1	2

*^a^The fingerprint typing based on BoxAIR and (GTG)5. ^b^The number of strains of the same type as the representative strain. R, breast milk sample; D, mother stool sample; X, infant stool sample; T, the strain from Uighur; N, the strain from Li.*

**FIGURE 3:**
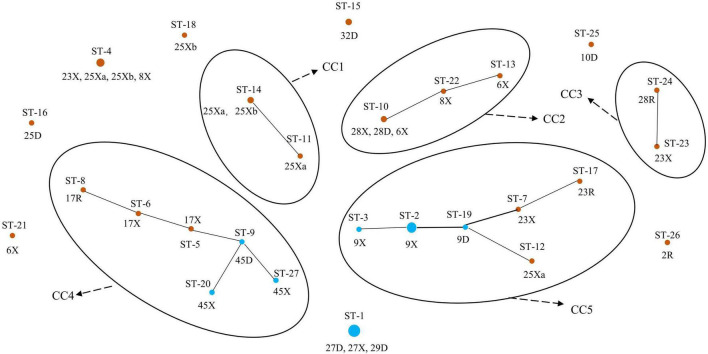
Population structure of 64 representative *L*. *paracasei* strains by goeBURST. Brown represents the Uighur; blue represents the Li.

We further defined the more closely related strains with similar allelic profiles as the same clonal complexes (CCs) (only one allele disparity), an entity including at least two or more STs in a set of strains investigated (Francisco et al., 2009; Nascimento et al., 2017). A diagram showing the origins and relationships of different STs are given in Figure 3. Thirteen of the 20 Uighur STs were incorporated into five CCs, and the remaining 7 STs could not be assigned to any group. Among the 7 STs of Li, 6 STs were classified into two CCs, and another one was a singleton. In particular, the STs from mother–infant pairs (28D-X, 23R-X, 17R-X, 45D-X, and 9D-X) or twins (25Xa and 25Xb) were more likely incorporated into the same CC. Overall, the 27 STs were classified into five CCs and eight singletons.

### Strain Relationships Based on Genetic Evolution of Sequence Types in Different Isolated Individuals and Ethnic Groups

**FIGURE 4:**
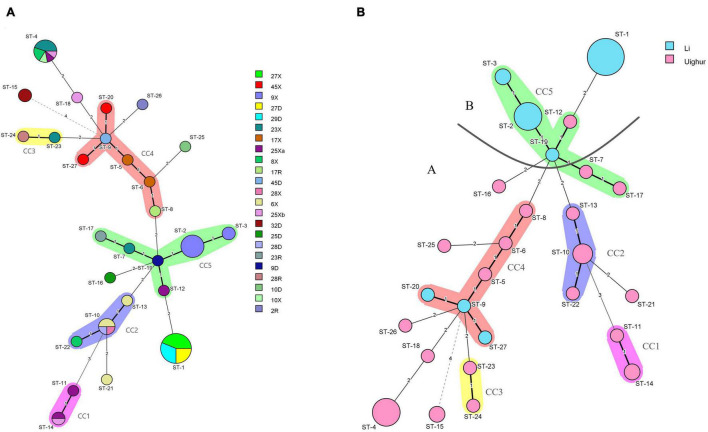
**(A)** Minimum-spanning tree analysis of 106 *L*. *paracasei* strains based on rep-PCR and multilocus sequence typing (MLST) date according to the individual. Different colors in the figure represent different sample sources. Each circle indicates a sequence type (ST); the size of the circle is proportional to the number of individuals sharing the same ST. **(B)** Minimum-spanning tree analysis of 64 representative *L*. *paracasei* strains based on MLST date according to region. Each circle indicates a ST; the size of the circle is proportional to the number of strains, and the type of line between isolates indicates the strength of the genetic relationship between these isolates (black line, strong relationship; gray line, intermediate relationship; dotted line, weak relationship), The different colors represent the strains isolated from different ethnic groups.

The minimum-spanning tree of STs for the different isolated individuals is shown in Figure 4A. We found that the different strains from the same host had similar STs (such as 27D, of which four strains were ST-1), most likely because these are closely related host species that possess similar niches in the gut (shared host glycan composition, diet composition, and host immune system). Notably, the hosts of 9X (ST-3, ST-2), 17X (ST-5, ST-6), and 6X (ST-10, ST-13) had more than one STs, suggesting that these strains were likely distributed in multiple ecological niches in the intestinal tract. Most strains from the same mother–infant pairs had similar STs and differed from each other by only one allele, while compared with the strains from non-mother–infant pairs, the largest difference was four alleles. For example, the disparity between the same mother–infant pair 45D (ST-9) and 45X (ST-20, ST-27) has only one allele but has four alleles difference when compared to 32D (ST-15) from non-mother–infant pairs. In addition, ST-1, ST-2, ST-10, ST-12, and ST-24 were found in the mother–infant pairs 27D-X, 9D-X, 28D-X, 28R-X, and 25D-Xa, respectively, suggesting vertical transmission. Particularly, there were two STs (ST-10, ST-24) that were found in mother–infant pair 28 (R, D, X). We found no cases where individuals from both ethnic groups shared a single ST.

In order to analyze the population structure of *L*. *paracasei*, a minimum-spanning tree of 27 STs was constructed based on Prim’s algorithm by BioNumerics v8.0 software. As could be seen from Figure 4B, the STs of Li formed major group B, while most of the STs from Uighur strains were clustered into major group A including four CCs (CC1, CC2, CC3, CC4). The ST-15, ST-11, and ST-14 from Uighur showed the farthest genetic relationship with other STs of major group A. Strains belonging to the same CC were relatively related clones. It is worthy of note that three STs from Li (ST-9, ST-20, and ST-27) were incorporated into CC4 consisting of strains of Uighur origin, indicating that the possibility that closely related strains were present in the two populations cannot be ruled out. Nevertheless, both the minimum-spanning tree and the concatenated sequence of the seven housekeeping genes (in total 2,940 bp) (Supplementary Figure 2) based on 27 STs of 64 strains reflected the ethnic origin of the strain well, despite some degree of “fuzziness.”

### Absence of Recombination Among *Lacticaseibacillus paracasei* Sequence Types

**FIGURE 5:**
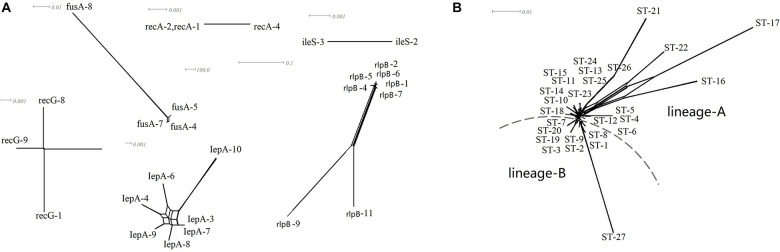
**(A)** Single housekeeping gene split tree diagram of *L*. *paracasei*, **(B)** split decomposition analysis of *L*. *paracasei* based on concatenate sequences of seven housekeeping genes.

A split decomposition analysis examining evidence for recombination among the 64 *L. paracasei* strains revealed different structures in the split graphs for all seven gene fragments. As shown in Figure 5, all the strains were divided into two distinct genetic lineages based on ethnic origin (lineage A from Uighur and lineage B from Li). The split-graph structures observed for only *lepA* and *rlpB* did not appear to exclude recombination phenomena, and the *phi*-test with *p*-values of 0.1074 and 0.4865, respectively, were not significant irrespective of the gene being considered. The *phi*-test result of seven housekeeping genes concatenated sequences was also not significant (*p* = 0), indicating that no genetic recombination occurs in all *L. paracasei* strains tested during evolution.

### Analysis of Carbohydrate Utilization by *Lactobacillus*

**FIGURE 6:**
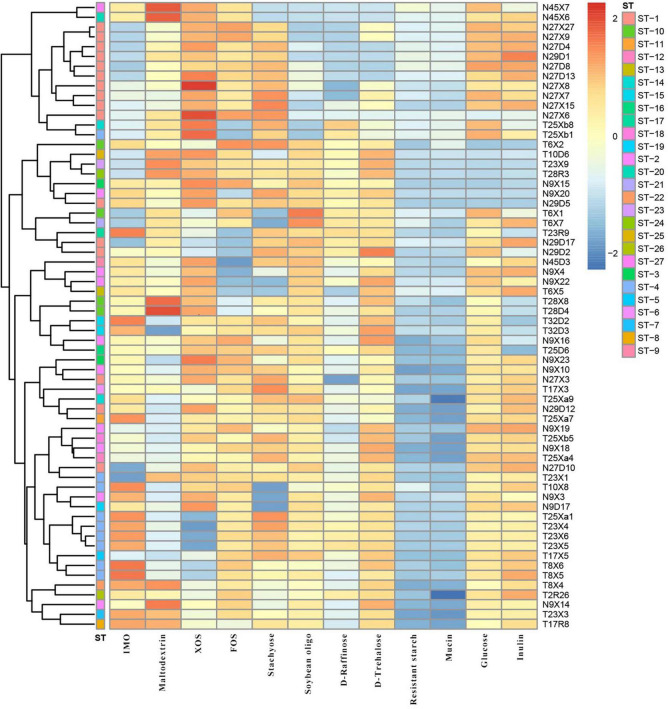
The cluster analysis of polysaccharide utilization and STs of 64 representative *L*. *paracasei* strains.

We evaluated the growth profiles of *L*. *paracasei* in 11 glycans by the growth OD_600_ value of the strains and divided the growth results into four groups: No growth (OD600 nm < 0.3), limited growth (OD600 nm = 0.3–0.5), moderate growth (OD600 nm = 0.5–0.8), and good growth (OD600 nm > 0.8). The carbohydrate substrates employed here included isomalto-oligosaccharide (IMO), maltodextrin, fructo-oligosaccharide (FOS), xylose-oligosaccharide (XOS), stachyose, soybean oligosaccharide, D-raffinose, D-trehalose, resistant starch, mucin, and inulin. As shown in Figure 6, strains of Li group preferred to metabolize stachyose, FOS, inulin, and XOS, while the Uighur strains had better utilization of IMO and soybean oligosaccharide. However, none of the strains grew well in the presence of mucin and resistant starch (OD600 nm = 0.3–0.5). Strains with the same STs had similar glycans utilization profiles; for example, the strains from (27D, 27X, 29D) were all belong to ST-1, and they were tended to cluster together, similar STs were ST-2, ST-4, and ST-10. More notably, *L. paracasei* isolates from the same ethnic group were more likely than strains from another to be incorporated into a specific phylogenetic clade with similar metabolic profile of glycan, revealing ethnically specific metabolic lineages to a large extent. As shown, T25xa1, T23 × 4, T23 × 6, T23 × 5, T17 × 5, T8 × 6, T8 × 5, T8 × 4, T2R26, T23 × 3, and T17R8 were clustered together, and they were all derived from Uighur. Furthermore, compared to the oligosaccharide utilization of strains on MLST scheme of *L. paracasei*, a genotype of strains was combined, revealing the existence of a link between them. We found that in the strains from the same ethnic group, not only STs but also glycans utilization characteristics cluster together to a large extent.

## Discussion

Accumulating evidence suggests that a variety of factors, including host genetic background, living environment, and especially dietary differences, may pose different challenges and selection pressures for human gut microbial contents at the microbiome level and individual bacterial group (Chaplinska et al., 2016; Kohl et al., 2018; Quan et al., 2021). Due to dramatic discrepancy in human lifestyle, selective action of long-term diet patterns may be the main reason that contributed to the difference of gut microbiota of ethnic groups located in different geographical areas (Beam et al., 2021; Couch et al., 2021). As is shown in Supplementary Table 1, we recorded in detail the main differences in diet between mother and infant pairs of the two ethnic groups of China during breastfeeding. The Uighur diet is characterized by animal-derived food, dried fruit, and dairy products, while the Li prefers a fruit-rich diet and seafood. In view of the significant differences in the metabolism of complex plant-derived polysaccharides in the diet and/or host-derived glycans by *Lactobacillus* spp., as well as the very different living environments of the two ethnic groups, it was expected that the composition and prevalence of *Lactobacillus* populations should differ significantly in the gut microbiota of the two ethnic groups.

As shown in Figure 1, *L. casei*, *L. paracasei*, *L. plantarum*, and *L. salivarius* were the most common species in mother–infant fecal samples, which was largely in agreement with other studies (Vaughan et al., 2005; Murphy et al., 2017; Zhang et al., 2020). However, for most milk samples, the strains isolated at the highest frequency were identified as *L. gasseri*, *L. oris*, *L. salivarius*, *L*. *fermentum*, and *L. paracasei*. It is worthy of note that most studies on human milk microbiome using next-generation sequencing (NGS) of specific 16S variable gene regions reported only their results at the taxonomic level of genus, as total bacterial loads of breast milk are much lower than in fecal samples (Fernández et al., 2013; Li et al., 2017; Murphy et al., 2017). Some studies have not even reported the presence of *Lactobacillus* spp. in breast milks during the first month postpartum, while for other studies using culture methods, *Lactobacillus* populations were not also detected in a considerable proportion of transitional and mature milk samples. Altogether, there were significant differences in the composition and abundance of *Lactobacillus* species between different study cohorts. For example, in some women’s milk samples in central Europe, the *Lactobacillus* species isolated most frequently were *L. salivarius* (35.00%), *L. fermentum* (25.00%), and *L. gasseri* (21.88%) (Soto et al., 2014). In a study on Egyptian human milk, *L. rhamnosus* was found to be the most frequently isolated species (20.8%), followed by *L. plantarum* (13.2%) and *L. casei* and *L. fermentum* (8.8%) (Mehanna et al., 2013). The results of the two studies mentioned above are inconsistent with our results. In contrast, our results coincided with the observation of Tannock (2002) and Kirtzalidou et al. (2011), who reported that the common *Lactobacillus* species isolated from breast milk and infant gut included *L. gasseri*, *L. paracasei*, *L. salivarius*, and *L. fermentum*, supporting the hypothesis that these lactobacilli species are likely to be persistent colonizers in the human gut (Tannock, 2002; Kirtzalidou et al., 2011). In our study, despite the difficulty and uncertainty of isolating *Lactobacillus* from breast milk, eight *Lactobacillus*, including *L. farciminis*, *L. casei*, *L. paracasei*, *L. fermentum*, *L. plantarum*, *L. salivarius*, *Lactobacillus acidophilus*, and *L. oris*, were each recovered in at least one mother–breast milk–infant triad. Among them, *L. paracasei* was the most prevalent species, with isolates retrieved in most, but not all, mother–breast milk–infant triads of the two ethnic groups, although less frequently isolated in breast milk.

Among all lactic acid bacteria (LAB), at least 10 species of *Lactobacillus*, including *L. paracasei*, have been shown to exhibit probiotic properties (Douillard and de Vos, 2019). However, the probiotic effect of *Lactobacillus* is not only strain- and species-specific but also closely associated with the adaptation of the bacterial strain screened to the gastrointestinal environment and daily diet of the host (Martino et al., 2018; Groussin et al., 2020). Due to their flexibility of lifestyle, including nomadic and free-living and ranging from free-living to symbiotic, most of the members of *Lactobacillus* in the human intestinal tract are not endosymbiont bacteria (Duar et al., 2017b). Nevertheless, many studies have shown that host-adapted lineages of *Lactobacillus* evolved from free-living ancestors, with these gut symbionts showing social host specificity (Powell et al., 2016; Mallott and Amato, 2021). For example, the former *L. reuteri*, as an excellent model symbiont organism, was found to display considerable genetic heterogeneity within the population and was diversified into host-specific lineages, implying a long-term association with particular vertebrate species (Frese et al., 2011; Duar et al., 2017a). More recently, in multiple studies on the gut microbiome of humans and great-apes, the long-term vertically transmitted bacterial endosymbionts, including *Bacteroides* and *Bifidobacterium*, were substantiated to retain hallmarks of co-diversification in their hosts, reflecting strain-level evolutionary history between bacteria and hosts (Moeller et al., 2016). However, little has been known about this symbiotic relationship and host specificity in the human gut to date. In our study, the predominant species *L. paracasei* shared by the two ethnic groups was selected to seek the ethnic specificity in the composition of *Lactobacillus* species and strain in different mother–infant cohorts. A goeBURST analysis revealed that a subset of *L. paracasei* isolates in each mother–infant pair (an average of four to six isolates per mother–infant pair) were identified to be monophyletic (maternal intestine, breast milk, and the corresponding infant’s intestine), confirming the co-occurrence of *L. paracasei* in mother–infant pairs. Despite the observation that *L. paracasei* isolates in some mother–infant pairs were of two different STs, due to only one allele disparity, these isolates were still considered as belonging to a very closely related clone, indicating that identical strains of the same species were transferred from the intestine of the mother to that of the infant by breastfeeding. Particularly, based on both rep-PCR and split decomposition and clustering analysis of MLST, *L. paracasei* strains derived from mothers and infants of multiple families of the same ethnic group tend to be incorporated into a specific phylogenetic clade or the same genotypic group (Figures 2–5).

In the present study, the habitats of mother–infant cohorts of two ethnic groups, including Xinjiang Uighur and Hainan Li of China, are separated by more than 4,000 km, with geographical and climatic environments being greatly different. The existing evidence suggests that gut microbes have limited dispersal between individual hosts that are geographically isolated. Theoretically, when considering several human sub-populations living in different geographical habitats with a large span, their symbionts experience very limited dispersal, leading to weak (or absent) gene flow between symbionts. Our analytic result did indicate that no gene recombination events were scarcely detected among lineages or genotypes consisting of *L*. *paracasei* strains from mother–infant cohorts of different ethnic groups. This result was coincidental to the fact that the two ethnic groups rarely intermarried for thousands of years. Although timescales of the geographic isolation in human populations are not long enough, this, together with significantly different diets between ethnic groups, can contribute to ethnic group specificity of individual bacterial taxa and divergence of the gut microbiome (Groussin et al., 2020). Therefore, similar to symbiont *L. reuteri* distributed in other vertebrates, it was not surprising that a specific lineage of *L. paracasei* possessing a distinctive metabolic profile of plant-derived polysaccharides combinations (Figure 6) is confined to a given dietary ethnic group. In fact, despite not being autochthonous members of the resident in human gut microbiota, *L*. *paracasei* was thought to rapidly adapt to intestinal ecosystems and has the potential to persist (Duar et al., 2017b). Naturally, a specific strain variant of bacteria adapted in response to its host population can be more beneficial to particular hosts compared to strains of other origin (Nishida and Ochman, 2019). So our results provide direct evidence for the existence of an individual bacterial lineage in the human gut associated with its human host population.

## Conclusion

In conclusion, based on culture dependence and *groEL* gene sequencing results, our findings demonstrated that the composition of *Lactobacillus* from Uighur were higher than that of Li at the species level. In addition, the results of MLST revealed that except for a few mother–infant pairs with more than one detected ST, most *L. paracasei* strains from the same mother–infant pair were monophyletic (same clone complex), clearly confirming vertical transmission of strains between infant and mother gut. More notably, *L. paracasei* isolates of the same ethnic group were more likely to be assigned as the same phylogenetic clade or CC with similar metabolic profile of glycan than from another ethnic group, supporting the hypothesis of ethnic specificity to a large degree. No gene recombination was found between the two ethnic groups, indicating that the two ethnic groups maintained a high degree of genetic purity in the process of evolution. So the probiotic characteristics of symbiotic microorganism are not only highly strain- and species-specific but also closely related to the myriad selection pressures of host luminal environment and dietary adaptation. Further studies are required to unveil the specific lineages of more ethnic groups, to get an insight in the causes of ethnic specificity based on complete genome sequencing, providing evidence for the development of personalized probiotic tailored to very homogenous localized populations from the perspective of maternal and child health.

## Data Availability Statement

The original contributions presented in the study are included in the article/Supplementary Material, further inquiries can be directed to the corresponding author/s.

## Ethics Statement

The studies involving human participants were reviewed and approved by the Ethics Committee of The First Affiliated Hospital, Shihezi University School of Medicine (2017-117-01). The patients/participants provided their written informed consent to participate in this study.

## Author Contributions

LY: formal analysis, methodology, validation, and writing—original draft. XZ: methodology and investigation. BL: resources. XL and FT: conceptualization the study. WY: conceptualization and supervision. YN: conceptualization, supervision, project administration, and funding acquisition. All authors contributed to the article and approved the submitted version.

## Conflict of Interest

The authors declare that the research was conducted in the absence of any commercial or financial relationships that could be construed as a potential conflict of interest.

## Publisher’s Note

All claims expressed in this article are solely those of the authors and do not necessarily represent those of their affiliated organizations, or those of the publisher, the editors and the reviewers. Any product that may be evaluated in this article, or claim that may be made by its manufacturer, is not guaranteed or endorsed by the publisher.

## Footnotes

1
http://blast.ncbi.nlm.nih.gov/
