# Supplementary material for: Ethnic Specificity of Species and Strain Composition of Lactobacillus Populations From Mother–Infant Pairs, Uncovered by Multilocus Sequence Typing
Source: Front Microbiol. 2022 Mar 4;13:814284. doi: 10.3389/fmicb.2022.814284 (PMC8979337; doi:10.3389/fmicb.2022.814284)
Supplement: Supplementary file 1 [file Data_Sheet_1.pdf]

Ethnic Specificity of Species and Strain Composition of *Lactobacillus* Populations from Mother-infant Pairs, Uncovered by Multilocus Sequence Typing

Lixia Yuan[1], Xuelin Zhang[1], Baolong Luo[1], Xu Li[1], Fengwei Tian[2], Wenli Yan[1]* **and** ***Yongqing Ni[1]****

[1]School of Food Science and Technology, Shihezi University, Fourth Nouth Ave., Shihezi 832000, Xinjiang, People's Republic of China, [2]School of Food Science and Technology, Jiangnan University, Wuxi, China

*Corresponding authors

Email addresses: Y. N.: niyqlzu@sina.com; W.Y.: wenliyan62@sina.com
Tell: Y.N.:15299950600; W.Y.:13095096273

Contents

Supplementary Figures... 3

Fig. S1 Fingerprint typing cluster of 64 representative strains of *L. paracasei* based on Box-air and (GTG)5 primers... 3

Fig. S2 The phylogenetic tree of 64 representative *L. paracasei* strains based on the concatenated sequence of the seven housekeeping genes. The maximum likelihood method was used for the analysis with 1,000 bootstrap repeats. 4

Supplementary Tables..5

Table. S1 The characteristics and demographic data of the mothers and infants in this study... 5

Table. S2 The survey overview of main diet of mothers and infants of two ethnic groups during breastfeeding in this study..5

Supplementary Figure

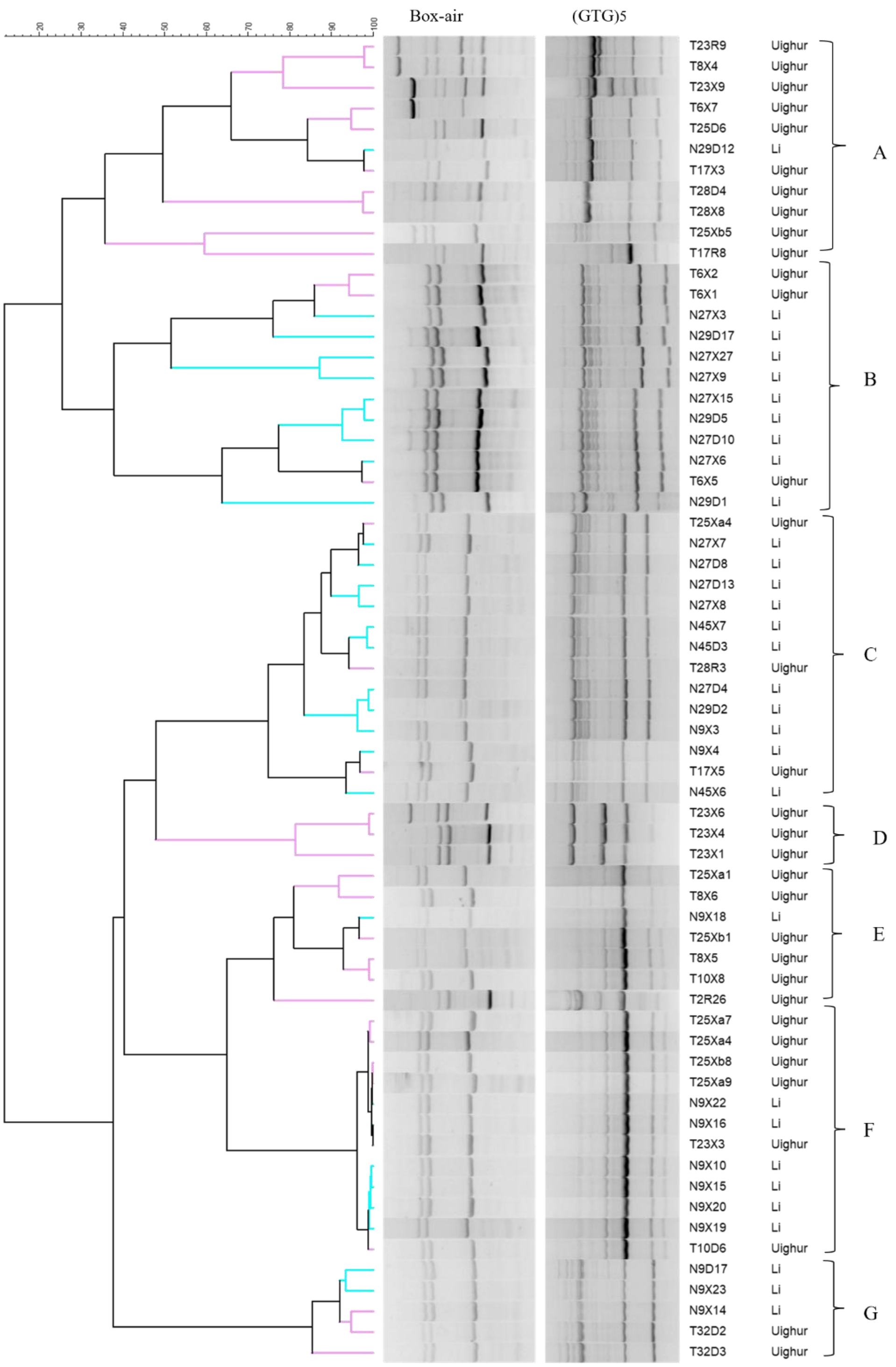

FIG. S1 Fingerprint typing cluster of 64 representative strains of *L. paracasei* based on Box-air and (GTG)5 primers.

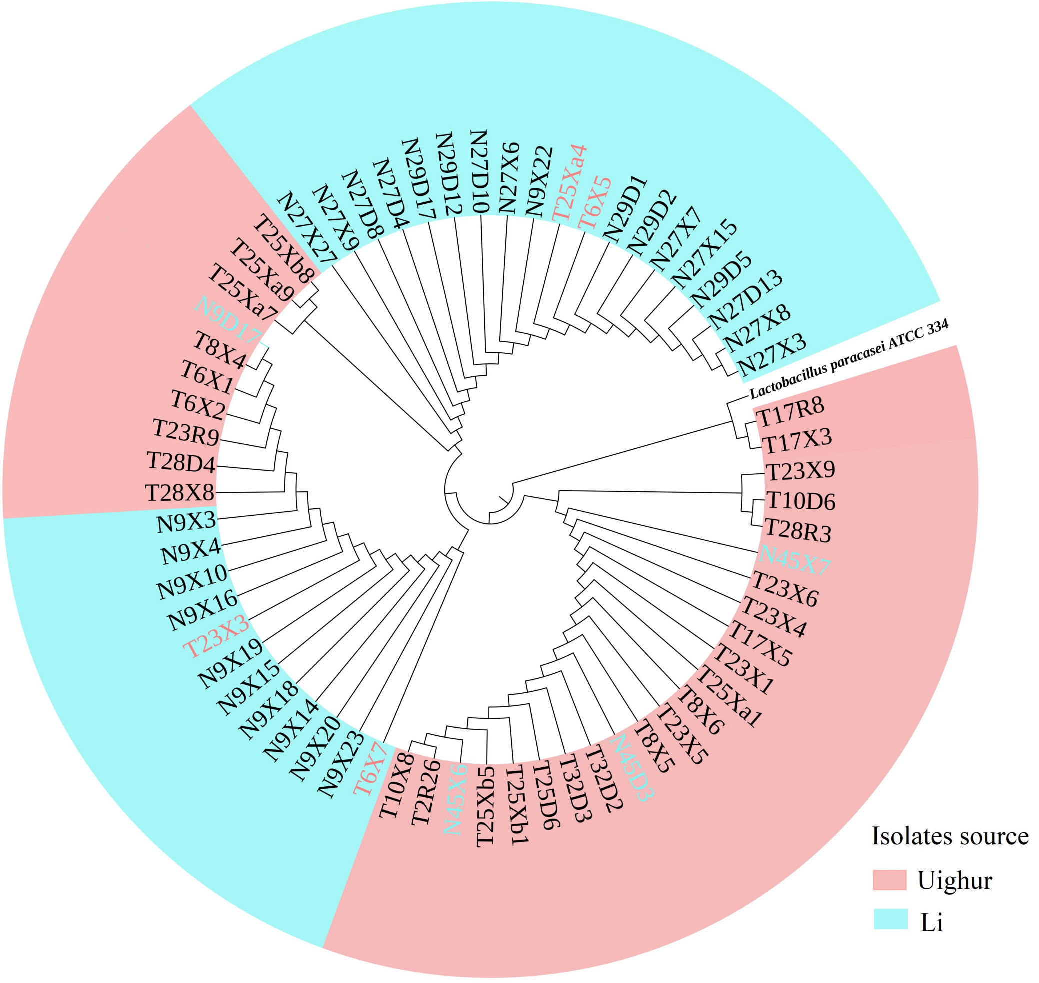

FIG. S2 The phylogenetic tree of 64 representative *L. paracasei* strains based on the concatenated sequence of the seven housekeeping genes. The maximum likelihood method was used for the analysis with 1,000 bootstrap repeats.

Supplementary Tables

Supplementary Table S1. The characteristics and demographic data of the mothers and infants in this study.

Characteristics and demographic data	Values (%)	
	Uighur	Li
**Infant sex**		
Male	8 (47)	7 (50)
Female	9 (53)	7 (50)
**Feeding way**		
Breastfeeding	0	1 (7)
breastfeeding + formula feeding	1 (6)	2 (15)
breastfeeding + liquid feeding	16 (94)	8 (57)
formula feeding + liquid feeding	0	3 (21)
infant weight（kg）	7.27 ± 0.07*	7.29 ± 0.57*
infant length（cm）	66	71
**Maternal BMI condition**		
Normal (18.5-23.9)	8	10
Slightly fat (24.0-26.9)	1	3
Obesity (27-29.9)	7	1
Unknown	1	0

*, *Mean* ± *SD*

Supplementary Table S2. The survey overview of main diet of mothers and infants of two ethnic groups during breastfeeding in this study

Ethnic groups		Main diet
**Li**	**Monther**	Breakfast: rice porridge, papaya porridge, powder soup (seafood) , occasionally pickles<br>Lunch: congee, sour melon fried meat, pickled cabbage, fish, snail, occasionally chicken and duck<br>Dinner: porridge<br>side dish: coconut , mango, papaya
	**Infant**	Feeding way: breast milk or milk powder
**Uighur**	**Monther**	Breakfast: milk tea, soy milk, milk, naan bread<br>Lunch: noodles, lamb and rice, beef, onion<br>Dinner: soup, rice noodles<br>side dish: dried fruit, rose sauce, cheese
	**Infant**	feedingway: Breast milk , milk powder, feed a liquid diet occasionally
